# Retraction Note: MicroRNA-1231 exerts a tumor suppressor role through regulating the EGFR/PI3K/AKT axis in glioma

**DOI:** 10.1007/s11060-026-05614-3

**Published:** 2026-05-28

**Authors:** Jiale Zhang, Jie Zhang, Wenjin Qiu, Jian Zhang, Yangyang Li, Enjun Kong, Ailin Lu, Jia Xu, Xiaoming Lu

**Affiliations:** 1 Department of Neurosurgery, The First Affiliated Hospital of Nanjing Medical University, 300 Guangzhou Road, Nanjing, Jiangsu Province 210029 People’s Republic of China; 2 Department of Neurosurgery, The Affiliated Hospital of Guizhou Medical University, 28 Guiyi Street Road, Guiyang, Guizhou Province 550004 People’s Republic of China; 3 Department of Emergency, Danyang People’s Hospital of Jiangsu Province, 2 Xinmin West Road, Danyang, Jiangsu Province 212300 People’s Republic of China

**Retraction Note: Journal of Neuro-Oncology (2018) 139:547–562** 10.1007/s11060-018-2903-8.

The Editor-in-Chief has retracted this article.

After publication, concerns were raised regarding some of the Western blot bands in Fig. 3A, Fig. 4B, and Fig. 6A, the bands in different treatments and different cells appeared to be highly similar.

The authors were contacted to provide an explanation and the raw data for these concerns but did not respond. The Editor-in-Chief therefore no longer has confidence in the presented data.

The authors have not responded to correspondence regarding this retraction.

